# Functional analysis of *Pogostemon cablin* farnesyl pyrophosphate synthase gene and its binding transcription factor PcWRKY44 in regulating biosynthesis of patchouli alcohol

**DOI:** 10.3389/fpls.2022.946629

**Published:** 2022-08-26

**Authors:** Xiaobing Wang, Yun Tang, Huiling Huang, Daidi Wu, Xiuzhen Chen, Junren Li, Hai Zheng, Ruoting Zhan, Likai Chen

**Affiliations:** ^1^Research Center of Chinese Herbal Resource Science and Engineering, Guangzhou University of Chinese Medicine, Guangzhou, China; ^2^Key Laboratory of Chinese Medicinal Resource From Lingnan (Guangzhou University of Chinese Medicine), Ministry of Education, Guangzhou, China; ^3^Joint Laboratory of National Engineering Research Center for the Pharmaceutics of Traditional Chinese Medicines, Guangzhou, China; ^4^College of Agriculture and Biology, Zhongkai University of Agriculture and Engineering, Guangzhou, Guangdong, China; ^5^Affiliated Cancer Hospital and Institute of Guangzhou Medical University, Guangzhou, Guangdong, China; ^6^Guangdong Food and Drug Vocational College, Guangzhou, China; ^7^Maoming Branch, Guangdong Laboratory for Lingnan Modern Agriculture, Maoming, Guangdong, China

**Keywords:** *Pogostemon cablin*, *PcFPPS*, *PcWRKY44*, biosynthesis, patchouli alcohol

## Abstract

Farnesyl pyrophosphate synthase (FPPS) plays an important role in the synthesis of plant secondary metabolites, but its function and molecular regulation mechanism remain unclear in *Pogostemon cablin*. In this study, the full-length cDNA of the FPP synthase gene from *P. cablin* (*PcFPPS*) was cloned and characterized. The expressions of *PcFPPS* are different among different tissues (highly in *P. cablin* flowers). Subcellular localization analysis in protoplasts indicated that PcFPPS was located in the cytoplasm. PcFPPS functionally complemented the lethal *FPPS* deletion mutation in yeast CC25. Transient overexpression of *PcFPPS* in *P. cablin* leaves accelerated terpene biosynthesis, with an ~47% increase in patchouli alcohol. Heterologous overexpression of *PcFPPS* in tobacco plants was achieved, and it was found that the FPP enzyme activity was significantly up-regulated in transgenic tobacco by ELISA analysis. In addition, more terpenoid metabolites, including stigmasterol, phytol, and neophytadiene were detected compared with control by GC-MS analysis. Furthermore, with dual-LUC assay and yeast one-hybrid screening, we found 220 bp promoter of *PcFPPS* can be bound by the nuclear-localized transcription factor PcWRKY44. Overexpression of *PcWRKY44* in *P. cablin* upregulated the expression levels of *PcFPPS* and patchoulol synthase gene (*PcPTS*), and then promote the biosynthesis of patchouli alcohol. Taken together, these results strongly suggest the *PcFPPS* and its binding transcription factor PcWRKY44 play an essential role in regulating the biosynthesis of patchouli alcohol.

## Introduction

*Pogostemon cablin* (*P. cablin*) is a perennial aromatic herb belonging to *Lamiaceae* family. From its Southeast Asia origins, *P. cablin* has been widely cultivated in southern China, including Guangdong, Guangxi and Hainan provinces, for over 1,000 years (Chen et al., 2013). Many parts of *P. cablin* plant, especially leaves and stem, are rich in numerous active components, such as sesquiterpenoids and flavonoids (Li et al., 2014) and alkaloids. Patchouli alcohol, a sesquiterpenoid obtained from the leaves of *P. cablin*, has been reported to relieve depression and stress (Zhuo et al., 2020), control appetite, and improve sexual drive (Swamy and Sinniah, 2015). Furthermore, patchouli alcohol shows anti-inflammatory (Lian et al., 2018), antibacterial (Xu et al., 2017), anti-nociceptive (Yu et al., 2019), and antifungal (Kocevski et al., 2013) properties.

Farnesyl pyrophosphate synthase (FPPS) is a key enzyme occupying the branch point of the mevalonate metabolic pathway (MVA). It contains five conserved domains, namely, domain I (GKXXR), domain II (EXXXXXXLXXDDXXDXXXXRRG), domain III (GQXXD), domain IV (KT) and domain V (GXXFQXXDDXXDXXXXXXXXGKXXXDXXXXK) and aspartic acid-rich region DDXXD in domains II and V (X represents any amino acid; Srivastava et al., 2008). FPPS catalyzes the condensation of one molecule of dimethylallyl diphosphate (DMAPP) and two molecules of isopentenyl diphosphate (IDP) to form FPP, the precursor of sesquiterpenoids, which is then used for synthesis of sesquiterpenoids with diverse structures in a reaction catalyzed by various sesquiterpene synthases (Szkopinska and Plochocka, 2005). Currently, *FPPS* genes have been identified in different plant species, such as *Withania somnifera* (Gupta et al., 2011), *Anoectochilus roxburghii* (Yang et al., 2020). These genes regulate plant development, secondary metabolism, and various biological processes, such as terpenoid and sterol biosynthesis. In recent years, an increasing number of studies have investigated the function of *FPPS* in plants. For example, *LeFPS1*, plays an important role in early development of plant organs in tomato (Gaffe et al., 2000). Transferring FPPS of modified signal peptide into *Artemisia annua* significantly increased the content of sesquiterpenoid artemisinin (Chen et al., 2000). In *Gynostemma pentaphyllum*, binding of transcription factor GpMYB81 to the promoter of *GpFPS1,* a key structural gene, activates its expression (Huang et al., 2021). In *Poria cocos*, the expression profile of *FPS* gene and content of total triterpenoids at different developmental stages indicated that the activity of *FPS* was positively correlated with the amount of total triterpenes produced by *P. cocos* (Wang et al., 2014). Although *FPP* synthase gene of *P. cablin* (*PcFPPS*) has been successfully cloned from *P. cablin* transcriptome library (Tang et al., 2019), its function has not been studied in detail.

It is well known that the expression levels of genes involved in plant metabolic pathways are usually regulated by transcription factors (TFs). Further, the interaction between TFs and gene promoters is considered the most common regulatory mechanism of gene expression. Common TFs include bHLH, MYB, and WRKY. For example, PatSWC4, an MYB-related transcription factor, promotes the biosynthesis of patchouli alcohol by directly binding to and activating the *PatPTS* promoter (Chen et al., 2020). WRKY transcription factor family is one of the largest TF families in plants that controls expression of genes involved in plant growth and development and secondary metabolic processes (Schluttenhofer and Yuan, 2015). For example, AaGSW2, a GST-specific WRKY transcription factor identified in *Artemisia annua*, is positively regulated by the direct binding of homeodomain proteins AaHD1 and AaHD8 to the L1 box of the *AaGSW2* promoter. Overexpression of *AaGSW2* in *A. annua* significantly increased GST density, whereas *AaGSW2* knockdown lines showed impaired GST initiation (Xie et al., 2021). Similarly, in *A. annua*, AaWRKY17 directly bound to the W-box motifs in the promoter region of the artemisinin biosynthetic pathway gene amorpha-4,11-diene synthase (*ADS*) and promoted its expression. Overexpression of *AaWRKY17* in *A. annua* decreases its susceptibility to *pseudomonas syringae* (Chen et al., 2021). In addition, 37 putative WRKY transcription factors with intact WRKY domains were identified in *Rehmannia glutinosa*, with overexpression of *RgWRKY37* increasing the content of acteoside and total phenylethanol glycosides (PhGs) in hairy roots (Wang et al., 2021). In our previous research, we conducted binding experiment of PcFPPS-Pro with total patchouli protein using DNA pull-down technology. The proteins annotated as TFs were selected from the pulled-down proteins, and the genes with the highest similarity to the candidate protein genes were screened from the patchouli transcriptome database (PRJNA528262) using the Basic Local Alignment Search Tool (BLAST). A total of seven genes were initially screened but four genes, including *PcWRKY44* that interacts with PcFPPS-Pro, were further screened using yeast one-hybrid technology (Y1H). Based on their gene expression profiles and previous results, we speculated that *PcWRKY44* is very likely involved in the biosynthesis of patchouli alcohol, but the exact mechanism remains unclear.

In this study, a survey and systematic characterization of the *FPPS* gene in *P. cablin* were carried out. A phylogenetic tree was constructed to test the evolutionary relationships. The expression profiles of *PcFPPS* were detected using reverse transcription-quantitative PCR (RT-qPCR) in different tissues of *P. cablin*. The subcellular localization of PcFPPS was tested in *Arabidopsis* protoplasts and the biological activity of FPPS enzyme was verified in *Saccharomyces cerevisiae* mutant strain CC25. Additionally, in functional characterization, transient overexpression of *PcFPPS* significantly increased the content of patchouli alcohol in pCAMBIA1304-*PcFPPS* leaves. Similarly, overexpression of *PcFPPS* significantly increased the content of phytol, neophytadiene, and stigmasterol in transgenic tobacco leaves. Furthermore, we identified a 220 bp *PcFPPS-pro* that can be bound by the nuclear-localized transcription factor PcWRKY44 using dual-LUC assay and Y1H assay. Overexpression of *PcWRKY44* significantly increased the content of patchouli alcohol in *P. cablin* leaves. Our results suggest that *PcFPPS* gene and its binding transcription factor PcWRKY44 play important roles in patchouli alcohol biosynthesis.

## Materials and methods

### Plant materials

The *P. cablin* plants used in this study were collected from Yangjiang City, Guangdong Province, China. Cuttage was used to obtain more *P. cablin* seedlings for analysis of expression levels of *PcFPPS* in leaf, stem, and flower tissues and patchouli alcohol content in leaves. Tobacco cultivar K326 (*Nicotiana tabacum*) was used for the genetic transformation experiments. *P. cablin* plants and transgenic tobacco plants were grown in a growth chamber at 25°C with a day/night cycle of 16/8 h light/dark. *Nicotiana benthamiana* seeds were kept in our laboratory and cultivated in an intelligent incubator with a 16/8 h photoperiod treatment and a 21°C/17°C day/night temperature for use in dual-luciferase reporter system assays.

### RNA extraction and gene isolation

Total RNA was isolated from tissues using the GeneMark plant RNA purification kit (GeneMarkBio, Taiwan, China). RNA concentration and purity were determined using spectrophotometer (IMPLEN GNBH, Germany). Oligo dT was used for cDNA synthesis. *PcFPPS* sequence was retrieved from our *P. cablin* PacBio transcriptome project (NCBI Accession no. PRJNA528262). Primer3Plus^1^ was used to design the cloning primers, when designing primers, multiple repeating bases should be avoided. In addition, the GC% content is preferably between 45 and 55%, the primer length is between 18 and 25 bp, and the Tm value is 40–60°C. The 3′ end is preferably C or G terminated. After primers were designed, Primer-BLAST in NCBI was used for primer validation. All primers used in this study were listed in Supplementary Table S1.

### Sequence feature analysis of *PcFPPS*

The molecular weight of PcFPPS was predicted on ExPASy. The localization of deduced protein was predicted on BaCelLo, whereas its transmembrane domains were analyzed on TMHMM. Conserved domains and signal peptide were analyzed using Pfam protein families database. Multiple sequence alignment was performed using DNAMAN. A phylogenetic tree was constructed by the neighbor-joining method using the MEGA7 software.

### Gene expression analysis

A HiScript®II qRT reagent kit with gDNA wiper (Vazyme, NanJing, China) was used to digest 400 ng of total RNA and residual genomic DNA samples, which were then reverse-transcribed. Reverse transcription-quantitative PCR (RT-qPCR) was performed on a real-time PCR system (Bio-Rad CFX96, CA, United States) with ChamQ Universal SYBR qPCR Master Mix (Vazyme, Q711-02/03) and gene-specific primers (Supplementary Table S1). RT-qPCR conditions were as follows: 95°C for 3 min for one cycle, followed by 40 cycles of 95°C for 10 s and 60°C for 30 s. The 18S rRNA of *P. cablin* served as an internal reference. Relative expression levels of the genes were calculated with the 2^-△△Ct^ method. Each sample was assayed in three independent biological replicates and three technical replicates. ANOVA and Student’s t-test were used to analyze the data.

### Subcellular localization

Complete ORFs of *PcFPPS* and *PcWRKY44* without a termination codon were inserted into the pAN580 vector driven by 35S promoter. *Arabidopsis thaliana* protoplast cells were isolated and transfected as previously reported (Cao et al., 2016). EGFP fluorescent signals were visualized using ZEISS LSM 800 with Airyscan (ZEISS, Germany). The empty vector pAN580, which has an EGFP-Fusion tag, was used as a negative control.

### Functional complementation of PcFPPS in yeast

*Saccharomyces cerevisiae* strain CC25, an ergosterol auxotrophic strain, was used to confirm the function of PcFPPS. A fragment containing the coding region of *PcFPPS* was PCR-amplified using specific primers pair (Supplementary Table S1) and cloned into the expression vector pESC-TRP to yield pESC-TRP-*PcFPPS*. pESC-TRP and pESC-TRP-*PcFPPS* plasmids were transformed into CC25 competent cells using PEG/LiAC and cultured with TRP deficient medium at 30°C for 2–3 d. Positive colonies were verified using PCR. CC25, CC25 + pESC-TRP, CC25 + pESC-TRP-*PcFPPS* transformants were diluted 1x, 100x, and 1,000x respectively, then plated on YPD medium and incubated at 30°C and 42°C, respectively, for 16 h, followed by 37°C for 2 days to observe the growth of yeast.

### Transient overexpression in *Pogostemon cablin* plants

The complete ORF fragment without stop codon of *PcFPPS* was cloned into pCAMBIA1304 vector tagged with GUS to form pCAMBIA1304-*PcFPPS* construct with two restriction enzyme sites: *NcoI* and *SpeI*. *Agrobacterium tumefaciens* strain GV3101 harboring the empty vector pCAMBIA1304 or binary vector pCAMBIA1304-*PcFPPS* was cultured at 28°C in LB liquid media until the OD_600_ of the culture reached ~0.8. *Agrobacterium* cells were harvested and resuspended in induction buffer and incubated for 3 h at 28°C before infiltration. Leaves of *P. cablin* were infiltrated with *Agrobacterium* suspension and maintained for 3 days under normal growth conditions. The untransformed *Agrobacterium* GV3101 was used as the control group. To determine expression efficiency, histochemical staining was performed on the infiltrated leaves to detect the GUS activity. Tissues were incubated using a GUS staining kit (SL7160, Coolaber, China) according to the manufacturer’s instructions. Leaf tissues near the infiltration point were collected and immediately frozen in liquid nitrogen for further analysis.

To evaluate transient overexpression of *PcWRKY44* in *P. cablin* leaves, full-length coding sequences of *PcWRKY44* were cloned into pJLTRBO to construct pJLTRBO-*PcWRKY44* with the restriction enzyme sites *PacI* and *NotI*. Empty vector pJLTRBO was used as a negative control. Preparation and infiltration of *Agrobacterium* GV3101 (pSoup-p19) was performed, as previously described (Chen et al., 2020).

### Development of *PcFPPS* transgenic tobacco lines

Recombinant vector pCAMBIA1304-*PcFPPS* was transformed into tobacco strain K326 with wild-type K326 as the control. Transformation of tobacco was performed following the infiltration method using *Agrobacterium tumefaciens* GV3101. Seeds were harvested and plated onto the selection medium containing hygromycin to identify transgenic plants. The presence of the appropriate DNA inserts was confirmed in these plants with PCR, whereas the transcription of *PcFPPS* was determined with RT-qPCR.

### FPP enzymatic activity assays of transgenic plants

Transformed tobacco leaves were fully homogenized in phosphate-buffered saline (PBS) using a homogenizer. Then, the leaf tissue homogenate was centrifuged at 2000–3000 rpm/min for 20 min at 4°C to collect the supernatant. FPPS enzymatic activity was determined using Plant ELISA Kit (MyBioSource, Inc. United States) following the manufacturer’s instructions.

### Analysis of volatile compounds in leaves using GC-MS

The content of patchouli alcohol in transiently overexpressed *P. cablin* leaves was detected as previously described (Chen et al., 2019).

A total of 0.4 g leaves of transgenic tobacco were ground frizzed in liquid nitrogen, extracted with 1.5 ml hexane ultrasonic for 30 min, and then heated in a 56°C water bath for 40 min. The sample was centrifuged at 12,000 rpm/min for 3 min, the supernatant was passed through 0.22 μm organic membranes, and the filtrate was transferred into new vials for GC-MS analysis using an Agilent 7890B Gas Chromatograph with 5977A inert Mass Selective Detector (Agilent, United States). The gas chromatograph was equipped with an HP-5MS capillary column (30 m × 0.25 mm × 0.25 μm film thickness). The oven temperature was programmed as follows: from 35°C (5 min hold) to 300°C at a rate of 12°C/min and hold 5 min at 300°C. NIST14/Wiley275 Mass Spectral Library was used for metabolite identification. To analyze transgenic tobacco metabolites, the relative quantification of volatiles was carried out using the external standard method with cyclohexanone as the external standard substance.

### Cloning of the *PcFPPS* promoter and dual-luciferase reporter assays

The promoter region of *PcFPPS* was cloned using PCR strategy with primers designed to flank known genomic sequence of *PcFPPS*. A 938 bp DNA fragment was obtained and cloned into pEASY-Blunt Zero vector for sequencing. For the dual-luciferase reporter assay, full-length coding sequence of *PcWRKY44* was cloned into pGreenII 62-SK vector as effectors, whereas *PcFPPS-pro* was recombined with pGreenII 0800-LUC vector as reporters. The Renilla *LUC* gene in pGreenII 0800-LUC was used as an internal control. Empty pGreenII 62-SK was used as the negative control for the effector. pGreenII 62-SK, effectors, and reporters were then transferred into *Agrobacterium* strain GV3101 (pSoup-p19). The reporter strain harboring *PcFPPS-pro*: LUC was mixed with *Agrobacterium* strains containing 62-SK effector or 62-SK: *PcWRKY44* at a ratio of 1: 10. The mixture was injected into 5-week-old *N. benthamiana* leaves using a 1 ml syringe. Firefly luciferase and Renilla luciferase were quantified after 4 days of infiltration with the dual-luciferase assay kit (Promega, United States). The transcriptional regulation ability of *PcWRKY44* was assessed based on the LUC to REN ratio. At least six independent experiments were conducted for each combination.

### Yeast one-hybrid assays

For the Y1H assay, *PcFPPS-pro* was cloned into the bait vector pHIS2 with the restriction sites *EcoRI* and *SacI*. pHIS2-*PcFPPS-pro* was then transformed into yeast strain Y187 competent cells to test for toxicity and autoactivation. The ORF of *PcWRKY44* was cloned and fused to pGADT7 digested with *EcoRI* and *BamHI* to construct the prey vector. Then, pHIS2-*PcFPPS-pro* and pGADT7-*PcWRKY44* were cotransformed into Y187 competent cells. In addition, pHIS2-p53 and pGAD53m were cotransformed into Y187 competent cells as the positive control, whereas pHIS2-*PcFPPS-pro* and the empty vector pGADT7 were cotransformed into Y187 as the negative control. The transformants were cultured on SD agar medium lacking tryptophan and leucine (SD/−Trp/−Leu) and positive clones were transferred to SD/−Trp/-His/−Leu plates with 3-AT and grown at different concentrations to verify positive interaction between *PcFPPS-pro* and PcWRKY44.

## Results

### Isolation of the *PcFPPS* full-length cDNA and expression analysis

**Figure 1:**
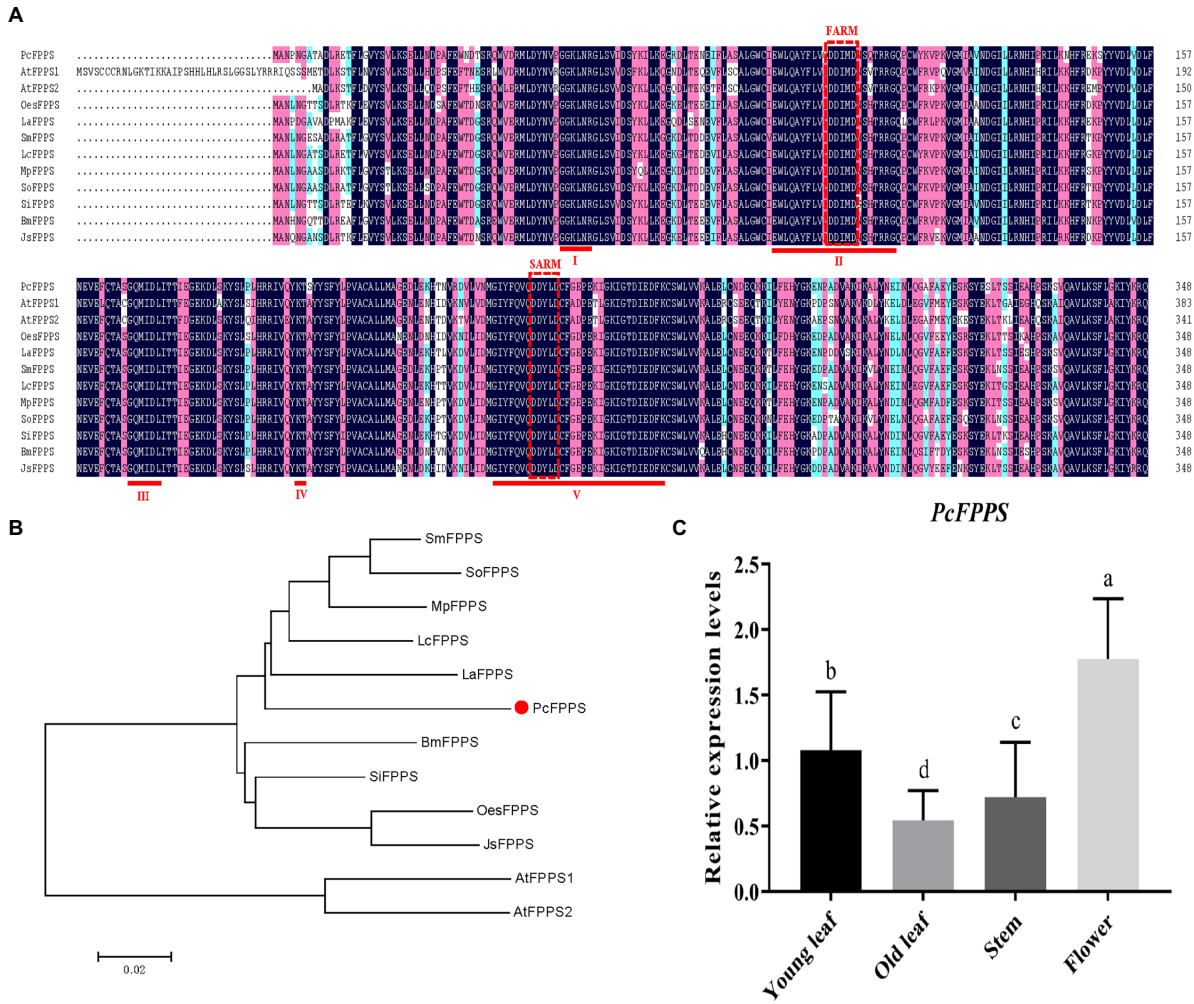
Bioinformatics analysis and expression profiles of *PcFPPS*. **(A)** Multiple sequence alignment of PcFPPS, *Arabidopsis thaliana* AtFPPS1 (NP_199588), *Arabidopsis thaliana* AtFPPS2 (NP_193452.1), *Salvia miltiorrhiza* SmFPPS (ABV08819.1), *Leucosceptrum canum* LcFPPS (ALT07952.1), *Mentha x piperita* MpFPPS (AAK63847.1), *Salvia officinalis* SoFPPS (AQY54371.1), *Sesamum indicum* SiFPPS (XP_011095887.1), *Bacopa monnieri* BmFPPS (ADV03080.1), *Lavandula angustifolia* LaFPPS (AGQ04160.1), *Olea europaea* var. *sylvestris* OesFPPS (XP_022879277.1), and *Jasminum sambac* JsFPPS (AIY24422.1). Similar, conserved and identical amino acid residues are shown in light blue, light purple, and dark blue. The five conserved regions contained in the FPPS family proteins are marked as I ~ V. Aspartate-rich regions located in conserved regions II and V are shown as red dashed boxes with FARM (First Aspartic Rich Motif) and SARM (Second Aspartic Rich Motif). **(B)** Phylogenetic tree of PcFPPS and other species FPPS proteins. The analysis was performed using the neighbor-joining method by the MEGA7. PcFPPS is shown as red solid dot. **(C)** The relative expression levels of *PcFPPS* in young leaf, old leaf, stem, and flower of *P. cablin*. Data are indicated as mean ± SD (*n* = 3). Statistically significant differences are analyzed using ANOVA combined with Bonferroni’s test (*p* < 0.05) and indicated as different letters.

PCR strategies were applied to isolate putative *PcFPPS* cDNA (Supplementary Figure S4) based on *P. cablin* PacBio transcriptome (NCBI Accession: PRJNA528262). Gene sequencing and sequence analysis revealed that *PcFPPS* contained an integrated open reading frame (ORF) with 1,050 bp, encoded 349 amino acid residues, and its estimated molecular weight was 40.11 kDa. Consistent with *FPPSs* from different plants (Wang et al., 2004), the deduced *PcFPPS* protein contains five highly conserved domains, two aspartate-rich motifs (DDxxD) FARM (First Aspartic Rich Motif) and SARM (Second Aspartic Rich Motif) located in conserved domains II and V, respectively. These motifs are considered the binding sites of IDP and DMAPP (Figure 1A). BaCeLo analysis predicted *PcFPPS* localized in the cytoplasm. TMHMM and Pfam protein families database analyses showed that *PcFPPS* lacks transmembrane domain and signal peptide, respectively. A phylogenetic tree illustrated that PcFPPS was highly homologous to *Lavandula angustifolia* LaFPPS (AGQ04160.1; Figure 1B).

The expression profiles of *PcFPPS* were investigated using RT-qPCR analysis. As shown in Figure 1C, all *PcFPPS* transcripts were detected in four different *P. cablin* tissues, with the highest level observed in flower, followed by young leaf, and lowest levels in stem and old leaf.

### Subcellular localization of PcFPPS in *Arabidopsis* protoplasts

**Figure 2:**
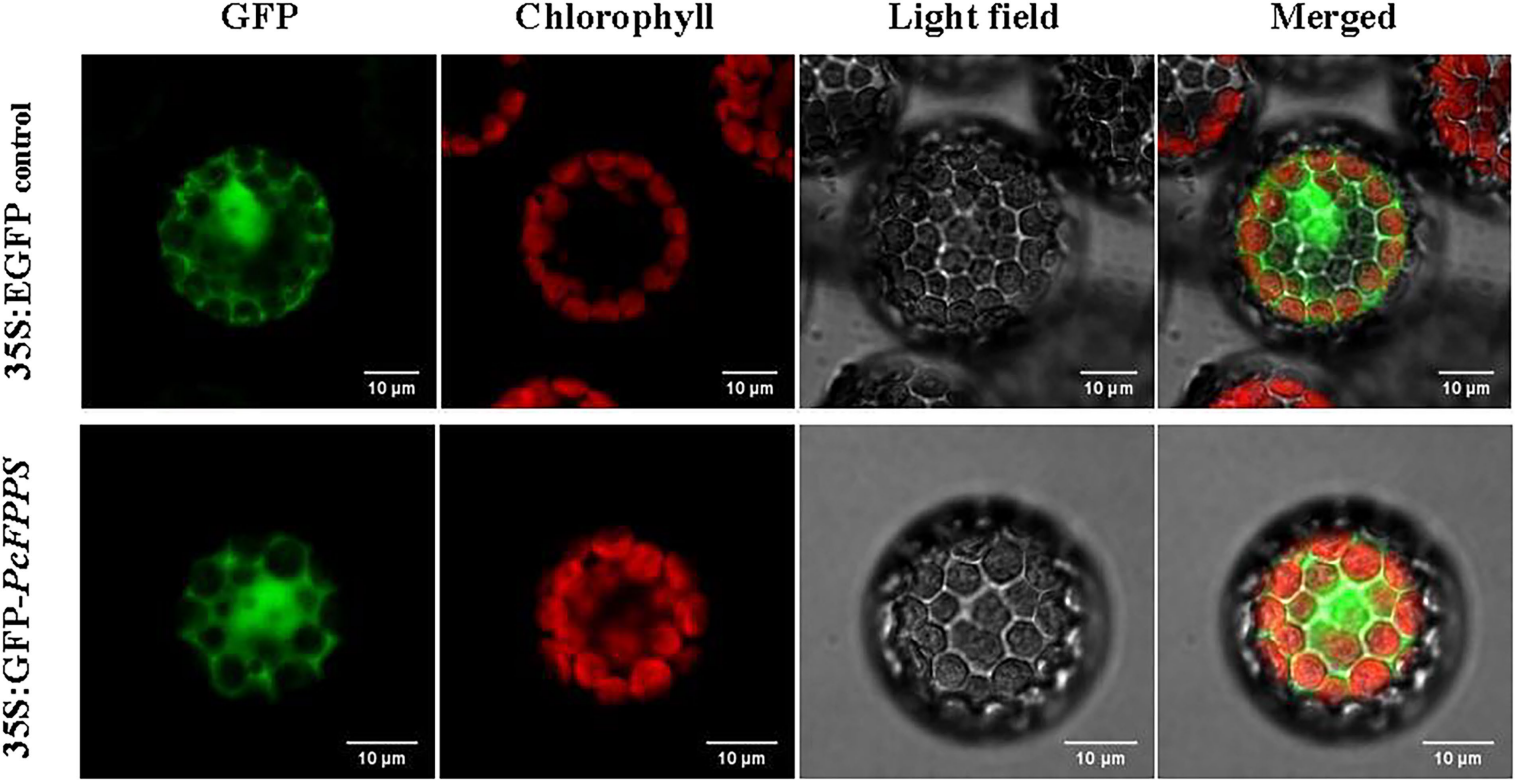
Subcellular localization of PcFPPS in *Arabidopsis* protoplasts. Control vector (35S:EGFP) and recombinant vector (35S:GFP-*PcFPPS*) are expressed in protoplasts of *Arabidopsis*. GFP, GFP fluorescence; Chlorophyll, Chlorophyll fluorescence; Light field; and Merged, superposition of fluorescence and light field. Bars, 10 μm.

*PcFPPS* was predicted with a high probability as localized in the cytoplasm using the BaCelLo database. To determine the subcellular localization of *PcFPPS*, the ORF of *PcFPPS* without a termination codon was inserted into the C-terminus of the GFP tag in vector pAN580. As shown in Figure 2, robust fluorescence was observed in the cytoplasmic compartments of *Arabidopsis* protoplasts expressing 35S: GFP-*PcFPPS*, suggesting that PcFPPS was localized in the cytoplasm.

### Functional complementation of *PcFPPS* in mutant yeast strain CC25

**Figure 3:**
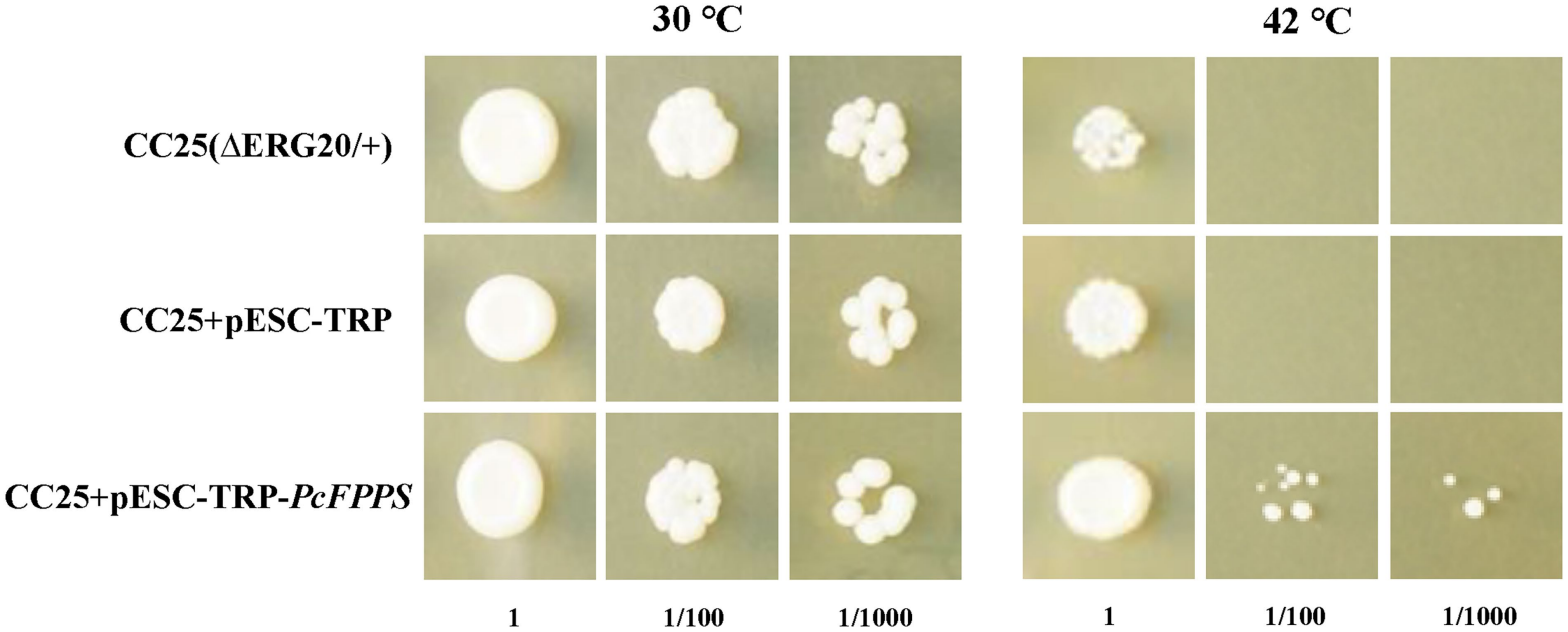
Functional complementation of PcFPPS in mutant yeast strain CC25 (MATa/MATalpha, Derg20/+). The growth testing and evaluation of strain CC25(∆ERG20/+), empty vector transformed CC25(CC25 + pESC-TRP) and pESC-*PcFPPS* transformed CC25(CC25 + pESC-TRP-*PcFPPS*) were carried out in parallel at appropriate temperature of 30°C and nonpermissive high temperature of 42°C for 16 h. Then the yeast growth was observed after culturing at 37°C for 2 days.

*S. cerevisiae* mutant strain CC25 (MATa/MATα, Derg20/+) is a sterol auxotrophic strain with two leaky mutations erg20-2 and erg12-2, which diminish the capacity of FPPS to condense GPP with IDP to produce FPP. In addition, CC25 is heat-sensitive and cannot grow at temperatures higher than 42°C. As shown in Figure 3, different dilutions of CC25, CC25 + pESC-TRP, and CC25 + pESC-TRP-*PcFPPS* transformants could grow at a suitable temperature (30°C for 16 h and then 37°C for 2 d), but CC25 and CC25 + pESC-TRP died at high temperature (42°C for 16 h and then 37°C for 2 days) after 100-fold dilution. By comparison, CC25 + pESC-TRP-*PcFPPS* could still grow on the plate, indicating that PcFPPS compensated for the functional defect of CC25 strain, and it encoded a biologically active enzyme that could catalyze the production of FPP.

### Transient overexpression of *PcFPPS* increases the accumulation of patchouli alcohol in *Pogostemon cablin* leaves

**Figure 4:**
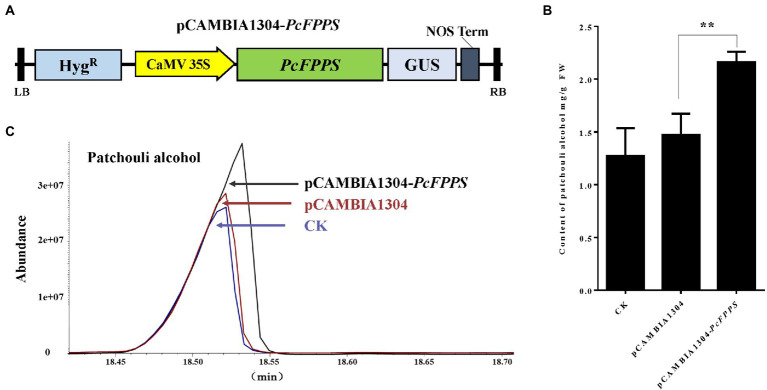
Transient overexpression of *PcFPPS* increases the accumulation of patchouli alcohol in *P. cablin* leaves**. (A)** The coding region without stop codon of *PcFPPS* was cloned into the pCAMBIA1304 vector controlled by the 35S promoter to form the pCAMBIA1304-*PcFPPS*. **(B)** Patchouli alcohol contents in leaves of CK (wild type), the empty vector pCAMBIA1304, and transformations pCAMBIA1304-*PcFPPS*. **(C)** Gas Chromatography–Mass Spectrometer (GC-MS) chromatograms of samples from the pCAMBIA1304-*PcFPPS* (top panel), pCAMBIA1304 (middle panel), and CK (bottom panel) leaves showing abundance of patchouli alcohol. Data represent the mean ± SEs of three biological replicates. The asterisks represent significant differences, as indicated by *t*-test (^**^p < 0.01). FW, fresh weight.

To further analyze the biological role of *PcFPPS* and its influence on the biosynthesis of patchouli alcohol, an efficient transient overexpression experiment was performed *in vivo* on *P. cablin* plants because designing a stable transformation system in this species is a challenge. The coding region without stop codon of *PcFPPS* was cloned into pCAMBIA1304 vector to generate pCAMBIA1304-*PcFPPS* construct, which was expressed under the control of the 35S promoter (Figure 4A). Subsequently, homologous transient overexpression was performed in the leaves of *P. cablin*. Leaf tissues were collected after *PcFPPS* was overexpressed for 3 days and used for RT-qPCR, GUS histochemical staining, and GC-MS analysis. *PcFPPS* transcript was significantly expressed (Supplementary Figure S1) in leaves after infiltration with *Agrobacterium* harboring overexpressed *PcFPPS* constructs. GUS histochemical staining was observed in leaf tissues infiltrated with empty vector pCAMBIA1304 and recombinant vector pCAMBIA1304-*PcFPPS* (Supplementary Figure S1). As shown in Figures 4B,C, overexpression of pCAMBIA1304-*PcFPPS* produced 47% more patchouli alcohol (2.16 mg/g) compared with the control group pCAMBIA1304 (1.47 mg/g). Altogether, these results indicated that *PcFPPS* could accelerate biosynthesis of patchouli alcohol in *P. cablin* (Figure 4).

### Overexpression of *PcFPPS* affects terpenoids metabolism in transgenic tobacco plants

**Figure 5:**
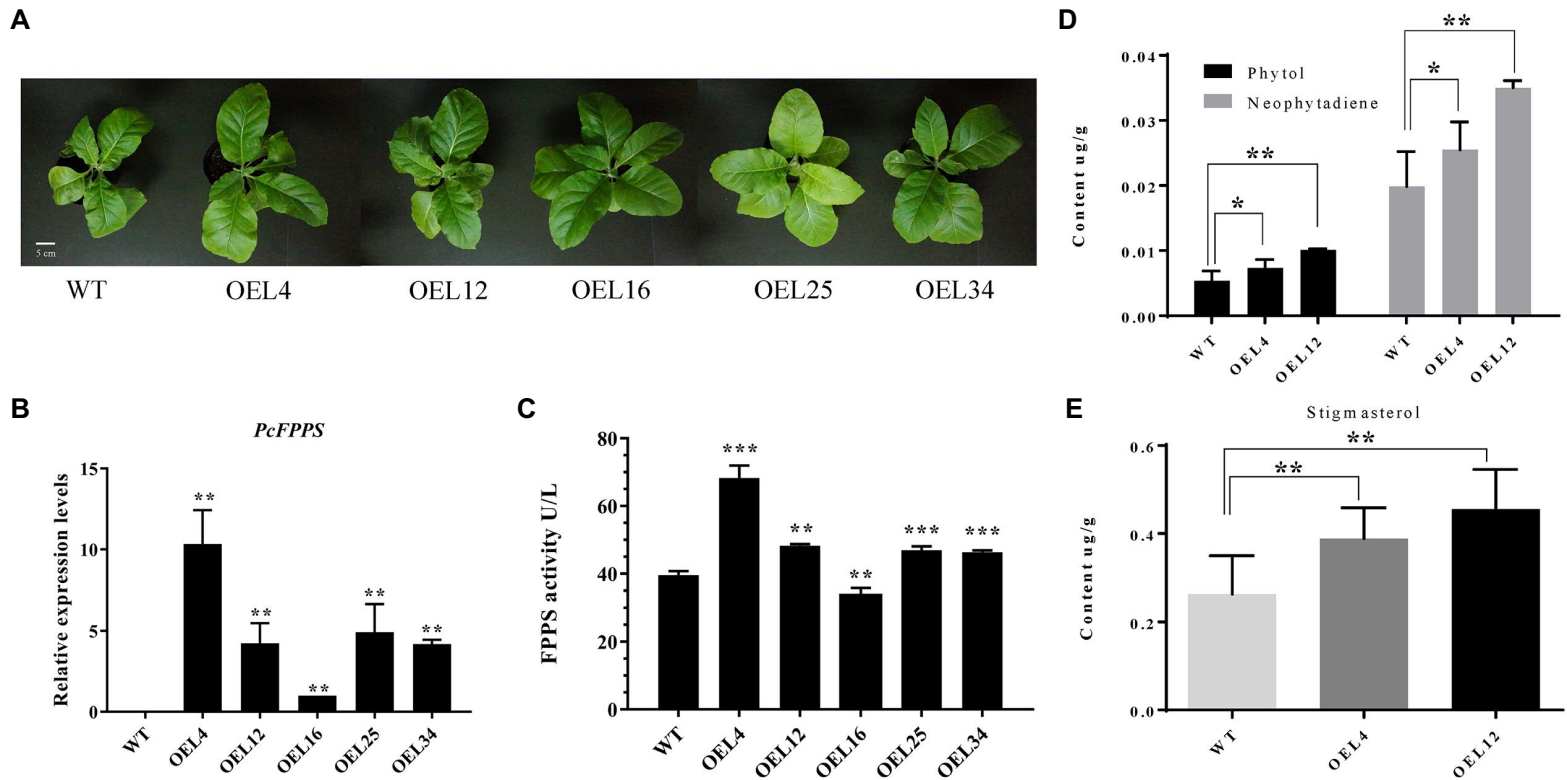
Overexpression of *PcFPPS* modulated the relative expression levels of *PcFPPS* and altered the endogenous terpenoid contents in transgenic tobacco plants. **(A)** Phenotypes of WT and transgenic tobacco plants. **(B)** Relative expression levels of *PcFPPS* in transgenic tobacco plants. **(C)** Activity of FPPS in WT and transgenic tobacco plants. **(D)** The content of phytol and neophytadiene detected in WT and transgenic tobacco OEL4 and OEL12. **(E)** The content of stigmasterol detected in WT, OEL4, and OEL12. Student’s *t*-test was performed to identify significant differences. One asterisk (^*^) indicates a significant difference (0.01 < *p* < 0.05) and two/three indicate a very significant difference (*p* < 0.01). Scale bars = 5 cm.

To further investigate the roles of *PcFPPS* in secondary metabolism and its effects on terpenoid production *in vivo*, a heterologous transgenic tobacco plant overexpressing *PcFPPS* was developed. PCR analysis using genomic DNA was performed to confirm the integration of *PcFPPS* gene into the transgenic tobacco lines. A total of 9 positive transgenic *PcFPPS* gene tobacco plants were obtained (Supplementary Figure S2). Figure 5A shows the phenotypes of wild-type (WT) and transgenic tobaccos used in this study. As shown in Figure 5A, all five *PcFPPS* transgenic tobacco lines appeared slightly larger than the WT. Subsequently, RT-qPCR was performed to verify the accumulation of *PcFPPS* transcript. As shown in Figure 5B, overexpression of *PcFPPS* upregulated *PcFPPS* transcripts in leaves of transgenic plants. The expression levels of *PcFPPS* in the OEL4, OEL12, OEL16, OEL25, and OEL34 lines were 1–10-fold higher than those of WT. ELISA showed that FPPS enzymatic activity was significantly upregulated in most overexpression lines compared with WT. Among them, FPPS activity in OEL4 was 1.7 times higher than that of WT (Figure 5C). Two transgenic plants, OEL4 and OEL12, were chosen for subsequent GC-MS analysis. As shown in Figure 5D, OEL4 and OEL12 transgenic plants exhibited significantly higher levels of phytol (diterpene alcohol) and neophytadiene (diterpene) compared with WT lines. The content of phytol in OEL4 and OEL12 were 1.37-fold and 1.85-fold higher than that of WT, respectively. In addition, the contents of neophytadiene in OEL4 and OEL12 were 1.28-fold and 1.76-fold higher than that of WT. Stigmasterol, one of the triterpenoid sterols, was also detected in transgenic plants. Its content in OEL4 and OEL12 was 1.48-fold and 1.73-fold higher than that of WT, respectively (Figure 5E). The GC-MS results were consistent with the gene expression and FPPS enzymatic activity changes, indicating that *PcFPPS* played a key role in the formation of farnesyl diphosphate, a precursor of several classes of essential metabolites, including terpenoids and sterols.

### *PcFPPS-pro* can be bound by transcription factor PcWRKY44

**Figure 6:**
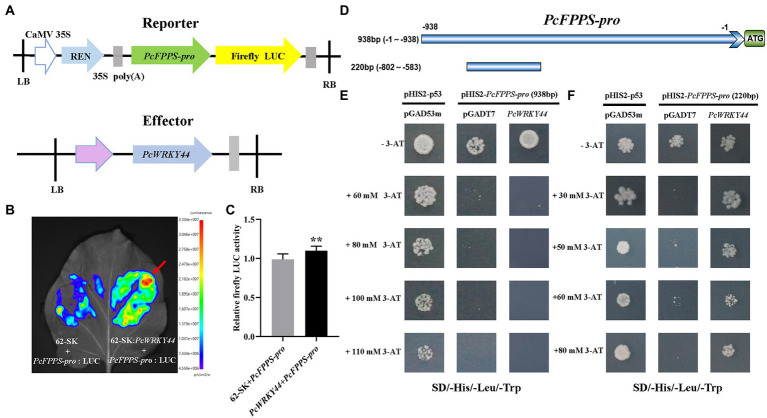
Dual-luciferase experiment and Yeast one-hybrid assay confirm that PcWRKY44 could bind to the PcFPPS-pro *in vivo* and *vitro*. **(A)** Schematic diagrams of the construction of reporter vector and effector vector. **(B)** Dual-LUC reporter imaging assay. *N. benthamiana* leaves were injected with the *Agrobacterial* GV3101-pSoup-p19 strains containing 62-SK + *PcFPPS*-*pro*:LUC and 62-SK:*PcWRKY44* + *PcFPPS*-*pro*:LUC. Arrow position indicates the strongest fluorescence. **(C)** Relative firefly LUC activity assay in tobacco leaves. Error bars are shown with six biological replicates. (Student’s *t*-test: ^**^ *p* < 0.01). **(D)** A schematic of *PcFPPS* promoter. The upper horizontal line represents the 938 bp promoter of *PcFPPS*, while the lower one represents 220 bp. **(E)** Yeast one-hybrid assays between the 938 bp *PcFPPS-pro* and PcWRKY44. Plasmids pHIS2-p53 and pGAD53m were cotransformed into Y187 as the positive control. **(F)** Yeast one-hybrid assays between the 220 bp *PcFPPS-pro* and PcWRKY44. Positive colonies indicated strong specific interactions between the 220 bp *PcFPPS-pro* and PcWRKY44.

To further analyze the function of *PcFPPS* in *P. cablin*, a 938 bp promoter region of *PcFPPS* was successfully cloned and sequenced using PCR method (Supplementary Figure S3). The transcription factor PcWRKY44 (Supplementary Figure S4) was obtained through DNA-pull down screening in the early stage of our research. To identify whether *PcFPPS-pro* (938 bp) can be bound by PcWRKY44, a Dual-LUC assay was performed in *N. benthamiana* leaves. *PcFPPS-pro* (938 bp) was cloned into vector pGreenII 0800-LUC vector as reporters and the ORF of *PcWRKY44* was cloned into vector pGreenII 62-SK vector as effectors (Figure 6A). A 5-week-old *N. benthamiana* was injected by *Agrobacterium* GV3101-pSoup-p19 cultures containing recombinant constructs (Figure 6B). *Agrobacterium* injection site containing 62-SK: *PcWRKY44* and *PcFPPS-pro*: LUC recombinant constructs showed a larger red area, revealing a strong interaction between PcWRKY44 and *PcFPPS-pro* in *N. benthamiana vivo.* It also suggested that this interaction is likely to exist in *P. cablin*. Relative firefly Luc activity was quantified after 4 days of infiltration using dual-luciferase assay. As shown in Figure 6C, the activity of *PcFPPS-pro* (938 bp) was significantly increased by PcWRKY44, suggesting that the expression of *PcFPPS* gene was activated by PcWRKY44.

Y1H assay was carried out to further investigate whether PcWRKY44 protein could directly bind to *PcFPPS-pro in vitro*. The *PcFPPS*-pro (938 bp) was firstly cloned into the bait vector pHIS2 and the full-length coding sequence of *PcWRKY44* was cloned into pGADT7. Y1H assays showed that the yeast cells with pHIS2-*PcFPPS-pro* (938 bp) and pGADT7-*PcWRKY44* could not grow on SD/−Trp/-His/−Leu plates, which was consistent with the negative control, suggesting that PcWRKY44 could not bind to the 938 bp *PcFPPS-pro* (Figure 6E). Subsequently, *PcFPPS-pro* was truncated to 220 bp (Figure 6D) and Y1H assay was performed again. As shown in Figure 6F, yeast cells with pHIS2-*PcFPPS-pro* (220 bp) and pGADT7-*PcWRKY44* could grow on SD/−Trp/-His/−Leu plates containing different concentrations 3-AT, which was consistent with the positive control, suggesting that 220 bp *PcFPPS-pro* can be bound by transcription factor PcWRKY44 to activate *HIS3* expression.

### PcWRKY44 located in the cell nucleus and could promote the accumulation of patchouli alcohol

The above results demonstrate that PcWRKY44 can bind to the 220 bp *PcFPPS* promoter and activate the transcriptional activity of genes downstream of the promoter, indicating that PcWRKY44 may play an important role in the biosynthesis of patchouli alcohol. To further analyze the function of PcWRKY44, subcellular localization and transient overexpression experiments were performed.

**Figure 7:**
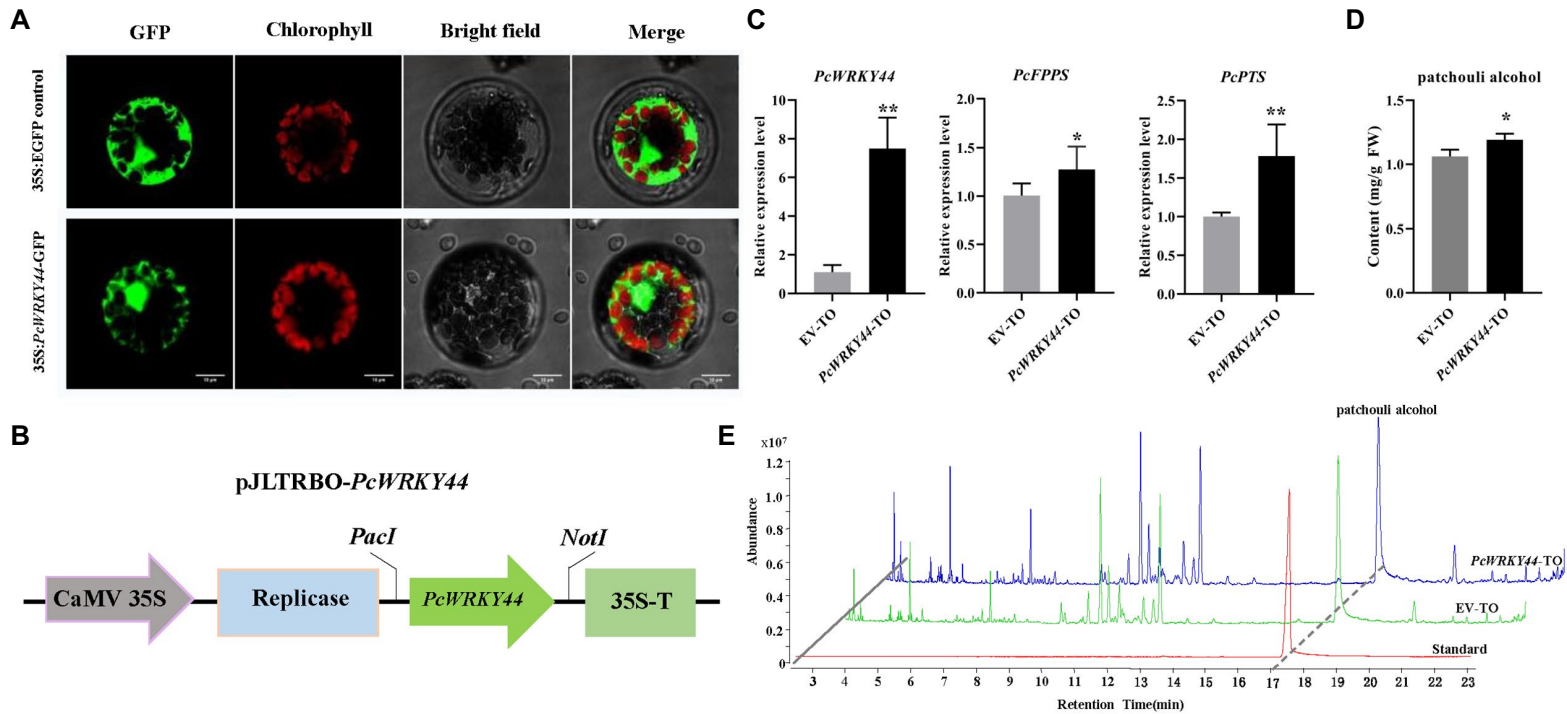
PcWRKY44 localized in the nucleus and transient overexpression of *PcWRKY44* increased the accumulation of patchouli alcohol in *P. cablin* leaves. **(A)** Subcellular localization analysis of the 35S:*PcWRKY44*-GFP fusion protein in *Arabidopsis* protoplasts. **(B)** Model of the pJLTRBO-*PcWRKY44* constructs. The ORF of *PcWRKY44* was cloned into the pJLTRBO vector with the restriction enzyme sites *PacI* and *NotI*. **(C)** The relative expression levels were analyzed by RT-qPCR for *PcWRKY44*, *PcFPPS,* and *PcPTS* in EV-TO and *PcWRKY44*-TO plants. **(D)** The content of patchouli alcohol detected in EV-TO and *PcWRKY44*-TO leaves by GC-MS. **(E)** GC-MS chromatograms of samples from the standard, EV-TO, and *PcWRKY44*-TO showing abundance of patchouli alcohol. Asterisks indicate a significant difference from the control (Student’s *t*-test; ^**^p < 0.01, ^*^p < 0.05). Error bars are shown with the three biological replicates.

The ORF of *PcWRKY44* without a termination codon was inserted into the N-terminus of the GFP tag in vector pAN580 for subcellular localization analysis. The 35S: *PcWRKY44*-GFP and 35S: EGFP constructs were separately introduced into *Arabidopsis* protoplast cells. The GFP fluorescence of the cells transformed with 35S: *PcWRKY44*-GFP was observed in the nucleus, indicating that *PcWRKY44* is a nuclear protein (Figure 7A).

The full-length coding sequence of *PcWRKY44* was cloned into pJLTRBO to construct pJLTRBO-*PcWRKY44* (Figure 7B). Subsequently, an *Agrobacterium*-infiltrated homologous transient overexpression assay in *P. cablin* was performed. In *PcWRKY44*-TO *P. cablin* leaves, the expression levels of *PcWRKY44, PcFPPS,* and *PcPTS* were all higher than that of EV-TO (Figure 7C). Patchouli alcohol content in *PcWRKY44*-TO and EV-TO was determined using GC-MS. Consistent with the gene expression profiles of *PcWRKY44, PcFPPS,* and *PcPTS*, patchouli alcohol accumulation was increased in *PcWRKY44*-TO. Transient overexpression of *PcWRKY44* produced levels of patchouli alcohol (1.19 mg/g FW) 12.3% higher than that of the EV-TO (1.06 mg/g FW; Figures 7D,E). The above results indicate that *PcWRKY44* could promote the biosynthesis of patchouli alcohol in *P. cablin*.

## Discussion

Hundreds of different drug products from *P. cablin* have been registered for use by the State Food and Drug Administration of China (SFDA), including various pills, 245 oral fluid agents, tablets, capsules, and granules (Chen et al., 2013). Therefore, *P. cablin* represents an effective herbal remedy with great application potential in diverse clinical situations. Patchouli alcohol is sesquiterpene alcohol with antibacterial, antifungal, and antiviral activity, isolated exclusively from patchouli oil. Patchouli alcohol also inhibits lipopolysaccharide-induced inflammation in the cells (Xian et al., 2011). However, there are considerable theoretical and technological lags limiting our understanding of the biosynthesis and regulatory mechanism of patchouli alcohol in *P. cablin*, which hinders sustainable pharmacological development and utilization of *P. cablin*. Our study systematically performed functional analysis of *PcFPPS* encoding farnesyl pyrophosphate synthase and revealed that PcFPPS activates terpenoids and patchouli alcohol biosynthesis in *P. cablin*. Moreover, PcWRKY44 was characterized and proposed as a critical transcriptional activator of *PcFPPS*. This study provides a detailed analysis of the roles of *PcFPPS* and *PcWRKY44* in patchouli alcohol biosynthesis, elucidating the functional role and transcription regulatory network of patchouli alcohol production.

FPPS in higher plants has been reported to play an important role in organ development and as a key regulatory enzyme involved in terpenoid biosynthetic pathways (Qian et al., 2017; Qin et al., 2018). To understand and elucidate the functions of *PcFPPS* in *P. cablin* terpenoids biosynthesis, we functionally confirmed PcFPPS through a yeast complementation assay and analyzed its function in *P. cablin* by transient overexpression and stable transformation expression in tobacco. The function of the *PcFPPS* gene was verified by functional complementation of mutant yeast strains lacking FPPS activity. *P. cablin* was transiently transformed with a construct harboring *PcFPPS* under the control of the 35S promoter. GC-MS analysis revealed that *PcFPPS* accelerated terpene biosynthesis, resulting in an ~47% increase in patchouli alcohol compared with the control (Figure 4). The results demonstrate that *PcFPPS* activated the terpene biosynthesis pathway and accelerated the accumulation of patchouli alcohol. With the overexpression of *PcFPPS* in tobacco, the transgenic tobacco appeared to grow larger and more vigorous than WT tobacco (Figure 5A). However, *Arabidopsis thaliana* overexpressing *FPS1S* showed a senescence-like phenotype (Masferrer et al., 2002), which was contrary to our findings, preliminarily indicating that *PcFPPS* may have a more unique function in *P. cablin* growth and development. However, further study is required to verify this function. In addition, the results of FPPS enzyme activity were significantly upregulated, and transgenic plants exhibited significantly higher levels of phytol, neophytadiene, and stigmasterol (Figure 5). Phytol is an acyclic diterpene alcohol molecule and a constituent of chlorophyll with a wide range of biological effects (Islam et al., 2018). Neophytadiene was identified as a natural diterpene herbal component isolated from flue-cured tobacco (Banožić et al., 2021) and has been tested for its analgesic potential. These results indicate that *PcFPPS* enhanced enzymatic activity and activated terpenoid biosynthesis in plant. Stigmasterol is a phytosterol, one of the most abundant phytosterols in nature, produced through the mevalonate pathway (Aboobucker and Suza, 2019). FPP is a precursor for sterol synthesis, which agrees with our findings that overexpressed *PcFPPS* significantly increases the accumulation of stigmasterol. Overexpression of *PcFPPS* in *P. cablin* and tobacco plants resulted in upregulation of terpene and phytosterol biosynthesis, which is consistent with previous findings (Keim et al., 2012; Yang et al., 2017). Our study shows that in *P. cablin* plants, *PcFPPS* plays a key role in the formation of isoprenoid end products such as terpenoids and phytosterols.

In previous studies, it was found that plants contain at least two *FPS* genes, *FPS1* gene that encodes a long isoform FPS1L which is targeted to mitochondria and a short isoform FPS1S, for which the localization has not been reported (Clastre et al., 2011). *CrFPS* (which is closely related to *FPS2*), isolated from the Madagascar periwinkle (*Catharanthus roseus*), was targeted to peroxisomes in *C. roseus* cells; however, the CrFPS protein was retained in the cytoplasm (Thabet et al., 2011). The PcFPPS isolated in this paper may be closely related to FPS2 based on sequence alignment analysis. Therefore, we explored whether PcFPPS is located in peroxisomes. However, we found that 35S: GFP-*PcFPPS* displayed a diffuse pattern of fluorescence throughout the cytoplasm (Figure 2), which differed from that of the punctate fluorescent signal characteristic of peroxisomal proteins. Our results are consistent with those reported previously with regard to the subcellular distribution of FPP enzymes involved in the early steps of plant isoprenoid biosynthesis. This finding provides a new perspective for characterizing the biosynthesis of terpenes and/or patchoulol. The genome of *P. cablin* has been reported so far, and there are at least four *FPPS* genes in the patchouli genome (Shen et al., 2022), which are consistent with our previous transcriptome analysis results (Tang et al., 2019). In this study, *FPPS3* in Tang’s paper was selected, and named *PcFPPS* for study, because it was expressed at a higher level in flowers and leaves than the other three genes. Our results suggest that *PcFPPS* plays an important role in the biosynthesis of terpenes. As members of the *FPPS* family, it is reasonable to believe that the other three *FPPS* genes also have significant effects on the biosynthesis of terpenes in *P. cablin*, which remains to be further studied.

Currently, the transcriptional regulation of functional synthase genes involved in the biosynthesis of patchouli alcohol is not well understood, and this limits its industrialization exploitation. The growth and development of *P. cablin* is regulated by transcription factors. For example, PatDREB, a nucleus-localized AP2/ERF TF, was identified as a transcription activator that binds to the promoter of *PatPTS* to positively regulate jasmonate-induced patchouli alcohol biosynthesis (Chen et al., 2022). In this study, the promoter of *PcFPPS* was identified for the first time, and the PcWRKY44 transcription factor was identified to bind to 220 bp *PcFPPS-pro* to increase the transcriptional activity of *PcFPPS* (Figure 6). WRKY transcription factors family, localized in the nucleus, is one of the largest transcription factor families in plants (Rushton et al., 2010). Numerous WRKY transcription factors involved in plant metabolism have been identified in different plant species. For example, subcellular localization analysis of *Osmanthus fragrans* showed that p35S::GFP OfWRKY7/38/95/139 was localized in the nucleus (Ding et al., 2020), which is consistent with our observation that PcWRKY44 was localized in the nucleus (Figure 7A). In *Oryza sativa*, OsWRKY45 played an important role in the initiation of diterpenoid phytoalexin biosynthesis after inoculation with *Magnaporthe oryzae* (Akagi et al., 2014). A total of 58 WRKYs were identified in *Andrographis paniculata* genome, and 7 of them, including ApWRKY01, ApWRKY08, ApWRKY12, ApWRKY14, ApWRKY19, ApWRKY20, and ApWRKY50 may participate in the biosynthesis of andrographolide (Zhang et al., 2020). Overexpression of the WRKY gene may enhance the expression of structural genes and promote accumulation of key secondary metabolites in plants. For example, in *Ophiorrhiza pumila*, OpWRKY2 was found to directly bind and activate the *OpTDC* gene involved in the camptothecin synthesis pathway. Moreover, overexpression of *OpWRKY2* resulted in a more than 3-fold increase in camptothecin levels (Hao et al., 2021). In this study, overexpression of *PcWRKY44* increased the expression of *PcFPPS* and *PcPTS*, thereby enhancing synthesis and accumulation of patchouli alcohol. Taken together, the present findings highlight the transcriptional regulation mechanisms involved in patchouli alcohol biosynthesis. They are expected to promote genetic engineering of patchouli alcohol for industrial production.

## Data availability statement

The datasets presented in this study can be found in online repositories. The names of the repository/repositories and accession number(s) can be found in the article/Supplementary material.

## Author contributions

LC, XW, YT, and HH conceived and designed the experiments. LC, YT, HH, XC, JL, HZ, and DW performed the experiments. YT, LC, HH, and DW analyzed the data. LC, YT, and XW wrote the manuscript. All authors contributed to the article and approved the submitted version.

## Funding

This research was supported by the Key-Area Research and Development Program of Guangdong Province, China (Grant no. 2020B020221001), Natural Science Foundation of Guangdong Province (Grant no. 2019A1515011542), National Natural Science Foundation of China (Grant no. 81803657), and Independent Research and Development Projects of Maoming Laboratory (2021ZZ004).

## Conflict of interest

The authors declare that the research was conducted in the absence of any commercial or financial relationships that could be construed as a potential conflict of interest.

## Publisher’s note

All claims expressed in this article are solely those of the authors and do not necessarily represent those of their affiliated organizations, or those of the publisher, the editors and the reviewers. Any product that may be evaluated in this article, or claim that may be made by its manufacturer, is not guaranteed or endorsed by the publisher.

## Footnotes

^1^ http://www.bioinformatics.nl/cgi-bin/primer3plus/

## Supplementary material

SUPPLEMENTARY FIGURE S4 Gene sequencing of PcFPPS and PcWRKY44.
