# Supplementary material for: Functional analysis of Pogostemon cablin farnesyl pyrophosphate synthase gene and its binding transcription factor PcWRKY44 in regulating biosynthesis of patchouli alcohol
Source: Front Plant Sci. 2022 Aug 26;13:946629. doi: 10.3389/fpls.2022.946629 (PMC9458891; doi:10.3389/fpls.2022.946629)
Supplement: Supplementary file 2 [file Table_1.DOCX]

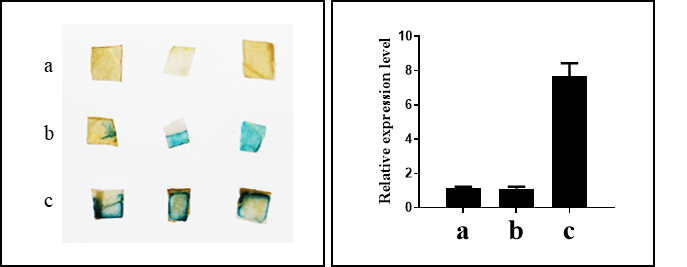


***PcFPPS***

**Figure S1. Histochemical analysis of GUS and expression levels of *PcFPPS* in transient transformed *P. cablin* leaves.** **(a)** Nontransformed plants as the negative control; **(b)** Transformed plants with the empty pCAMBIA1304 plasmid as the positive control; **(c)** Positive lines transformed with pCAMBIA1304-*PcFPPS*.


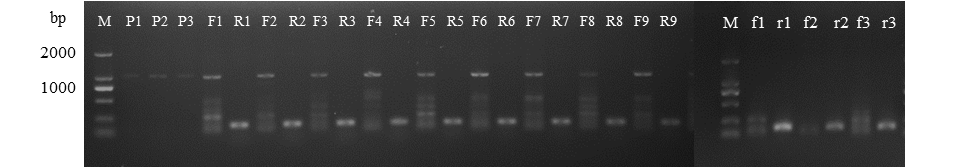


**Figure S2. PCR validation of *PcFPPS* in overexpressed transgenic tobacco lines.** M: DL 2000 DNA Ladder; P1-P3: PCR with genomic DNA of WT *P. cablin* and *PcFPPS* gene primers；F1-F9: PCR with genomic DNA of transgenic tobacco and *PcFPPS* gene primers; R1-R9: PCR with genomic DNA of transgenic tobacco and tobacco tRNA primers; f1-f3: PCR with genomic DNA of WT tobacco and *PcFPPS* gene primers; r1-r3: PCR with genomic DNA of WT tobacco and tobacco tRNA primers.


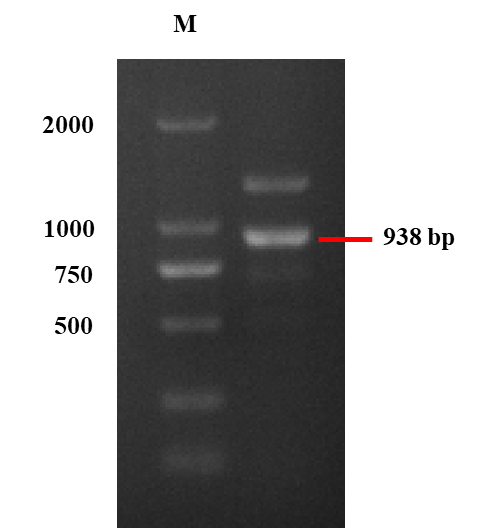


**Figure S3.** **Cloning of *PcFPPS* promoter.** gDNA of *P.cablin* was used as template.

**Table S1.** Primers used for cloning, RT-qPCR, and constructs generation.

| **Primers** | **Sequence (5’-3’)** |
| --- | --- |
| PcFPPS-F | ATGGCGAATCCGAACGGAGC |
| PcFPPS-R | TTTCTGTCTCTTGTAAATCTTGCCC |
| PLB-PcFPPS938-F | CCAAAGTTGACTGAGTGTTCCTT |
| PLB-PcFPPS938-R | CGCCCAGCAAGAATTCC |
| PcWRKY44-F | ATGGGTGAGGAGGATTTAGGC |
| PcWRKY44-R | TCATGCTGCAATCTGTTCTTCT |
| PAN-PcFPPS-F | tatacagatcttaaagcggccgcATGGCGAATCCGAACGGA |
| PAN-PcFPPS-R | aaatgtttgaacgatctgcagTTTCTGTCTCTTGTAAATCTTGCCC |
| PAN-PcWRKY44-F | aggacagcccagatcactagtATGGAGATTGGCCCTTATCTTTT |
| PAN-PcWRKY44-R | gcccttgctcaccatggatccACGAGCACCATTCCCAACAC |
| pESC-PcFPPS-F | ttgtaatccatcgatactagtTTTCTGTCTCTTGTAAATCTTGCCC |
| pESC-PcFPPS-R | aatttttgaaaattcgaattcATGGCCAATCCGAACGGA |
| 1304-PcFPPS-F | acgggggactcttgaccatggATGGCGAATCCGAACGGA |
| 1304-PcFPPS-R | aagttcttctcctttactagtCTGTCTCTTGTAAATCTTGCCC |
| 0800-PcFPPS-F | gtcgacggtatcgataagcttCCAAAGTTGACTGAGTGTTCCTTAA |
| 0800-PcFPPS-R | atctccaccgcggtggcggccgcCGCCCAGCAAGAATTCCG |
| 62-sk-PcWRKY44-F | aggacagcccaagctgagctcATGGAGATTGGCCCTTATCTTTT |
| 62-sk-PcWRKY44-R | gataagcttgatatcgaattcTTAACGAGCACCATTCCCAAC |
| pHIS2-PcFPPS938-F | gactcactatagggcgaattcCCAAAGTTGACTGAGTGTTCCTTAA |
| pHIS2-PcFPPS938-R | gattcgcgaacgcgtgagctcCGCCCAGCAAGAATTCCG |
| pHIS2-PcFPPS220-F | gactcactatagggcgaattcTACATCACCTATTCCAACCTTATACTTCA |
| pHIS2-PcFPPS220-R | gattcgcgaacgcgtgagctcTTACAGCGTTTTAATGTATAGTAAATTAAAAT |
| AD-PcWRKY44-F | gccatggaggccagtgaattcATGGAGATTGGCCCTTATCTTTT |
| AD-PcWRKY44-R | cagctcgagctcgatggatccTTAACGAGCACCATTCCCAAC |
| PJL-PcWRKY44-F | ttcgtgttcttgtcattaattaaATGGAGATTGGCCCTTATCTTTT |
| PJL-PcWRKY44-R | tcaagttgcaggaccgcggccgcTTAACGAGCACCATTCCCAAC |
| qPCR-Pc18S-S | TCGCCGTTCGGACCAAATAA |
| qPCR-Pc18S-AS | CGATGGTTCACGGGATTCTGC |
| qPCR-PcFPPS-F | TGCCACTTCACCGTCGTATT |
| qPCR-PcFPPS-R | TTTCAGGCTCACCGAAGCAA |
| qPCR-PcWRKY44-F | CGCAGTCATAAGGCCAAGGA |
| qPCR-PcWRKY44-F | TTAGCCACGAGAGGAACAGC |
| qPCR-PcPTS-F | TGGGTGCTGCTTCTCGTCCTC |
| qPCR-PcPTS-R | TGCGTTGTGGACTTGTTTCG |
| PCR-PcFPPS-F | ATGGCGAATCCGAACGGAGC |
| PCR-PcFPPS-R | TTTCTGTCTCTTGTAAATCTTGCCC |
| tRNA-F | CGAAATCGGTAGACGCTACG |
| tRNA-R | TTCCATTGAGTCTCTGCACCT |
