# Supplementary material for: Functional analysis of Pogostemon cablin farnesyl pyrophosphate synthase gene and its binding transcription factor PcWRKY44 in regulating biosynthesis of patchouli alcohol
Source: Front Plant Sci. 2022 Aug 26;13:946629. doi: 10.3389/fpls.2022.946629 (PMC9458891; doi:10.3389/fpls.2022.946629)
Supplement: SUPPLEMENTARY FIGURE S4 — Gene sequencing of PcFPPS and PcWRKY44. [file Data_Sheet_1.PDF]

Re: 946629 (Frontiers in Plant Science)

Dear Editors and Reviewers:

We would like to take this opportunity to express our sincere thanks to the editors and reviewers who raised a number of valuable comments and useful suggestions on our manuscript entitled "Functional analysis of *Pogostemon Cablin* farnesyl pyrophosphate synthase gene and its binding transcription factor PcWRKY44 in regulatory of the biosynthesis of patchouli alcohol". (Manuscript ID: 946629)

We also thank you a lot for allowing us to resubmit a revised copy of the manuscript. Your comments and those of the reviewers were highly insightful and enabled us to greatly improve the quality of our manuscript.

Based on your email, we have taken into consideration of the reviewer's comments and suggestions when revising the manuscript. Please see our point to point responses to all your comments below, and the corresponding revisions in the body of manuscript.

Looking forward to hearing from you.

Yours sincerely,

Dr. Likai Chen
Research Centre of Chinese Herbal Resource Science and Engineering
Guangzhou University of Chinese Medicine
Higher Education Mega center, Panyu, Guangzhou
P. R. China 510006
Email address: chenlk@gzucm.edu.cn
Tel: 86-020-39356150

Response to Reviewer 3 Comments

Sorry for my persistence. I know that you have do a lot of works, but we should make sure that the gene you studied is a pool of gene loci or a single locus. So, I still have some questions:

(1) The conclusion in your previuos round of response is that the primers for FPPS and PcWRKY44 are locus-specific, according to the electrophoresis of PCR amplification. In my opinion, a single clear band of electrophoresis of PCR amplification could not demonstrated it's specific, unless the sanger sequencing of the PCR amplicon is single one seuqence, as some paralogs generated recently could have every similar (even same) nucleotide length but the sequences are different.

<u>Response (1)</u>**:** Thank you very much, and we agree with your suggestion. In our study, sanger sequencing of PCR amplicon of *FPPS* and *PcWRKY44* CDS revealed that both genes were single one sequence. Please see the sanger sequencing results we upload in 'Review supporting file'.

In the revised manuscript, the sanger sequencing of the PCR amplicon was provided as Figure S4.

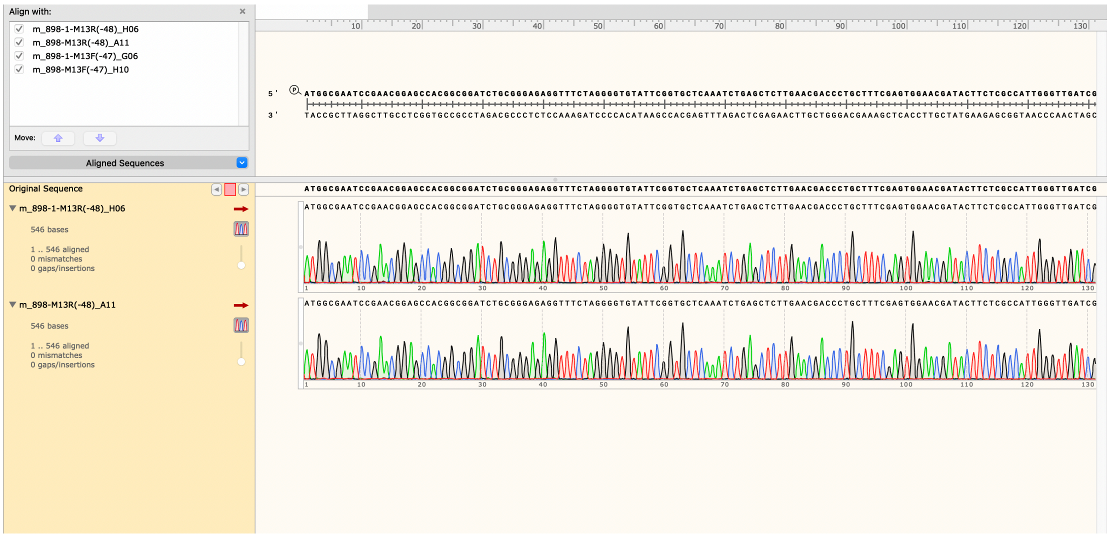

High quality sequencing signal and consistency indicates that the gene clone is single one sequence

(2) For the primers design for the FPPS genes, you said that you used the Primer3Plus. As far as I known, this software only give you a set of potential primers based on the template you provide, it does not consider whether the primer pair is specific to the whole genome. So, you must some additional processing steps to guarantee the specificity of the primers, please add these methods to the methods of the paper.

Response (2)**:** Thank you for bringing this point in our attention. In order to ensure the specificity of primers, we set some parameters when designing primers on the web, and the specific steps have been added to line 127-138 in the revised manuscript.

(3) You selected only one single locus of the four FPPS loci to study, although it has higher expression level than the others, it does not mean that the other loci have no significant influence on the terperne biosynthesis, unless you have some additional evidences. So, you should add some sentences in the discussions and conlusions of the paper to show these limitations to the readers and research community.

Response (3)**:** Thanks for your suggestion. In the revised manuscript, we have supplemented the description in the Discussion (lines 421-428), please see the revised manuscript for details.

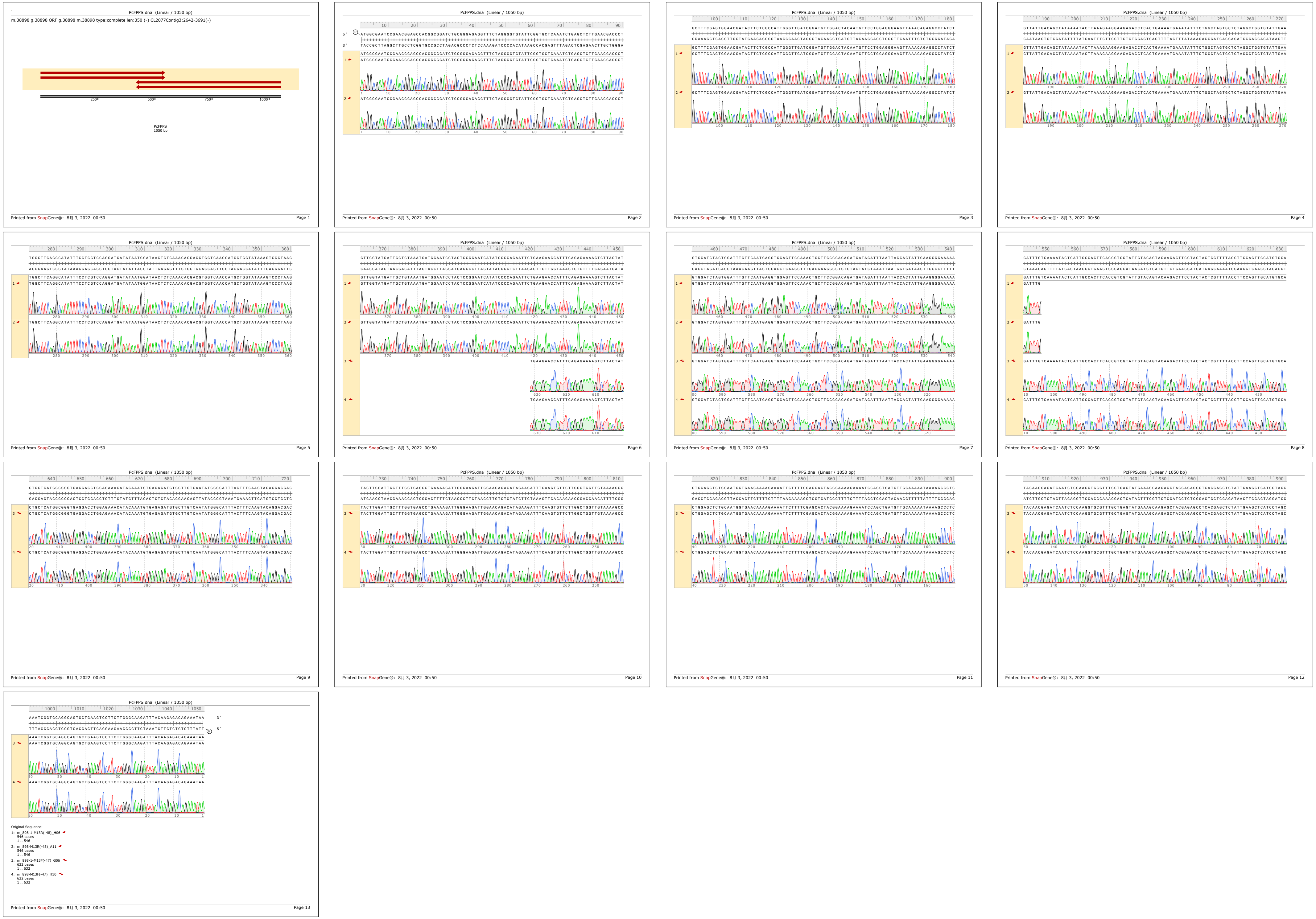

Figure S4. Gene sequencing of PcFPPS and PcWRKY44.

Sequence: PcWRKY44.dna (Linear / 2027 bp)
Enzymes: Unique 6+ Cutters (24 of 653 total)
Primers: 1 total

Unique Cutters Bold

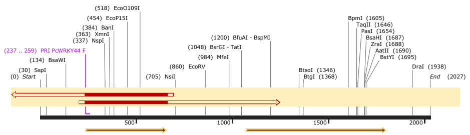

PcWRKY44
2027 bp

[illegible]

[illegible]

[illegible]

[illegible]

[illegible]

[illegible]

[illegible]

[illegible]

[illegible]

[illegible]

[illegible]

[illegible]

1990 2000 2010 2020

End (2027)

```
CTGTGCATCATATCTTCGACTTCGCCCCAATACATTCATATGATTCT      3'
GACACGTAGTATAGAAGCTGAAGCGGGGTTATGTAAGTATACTAAGA  (P) 5'
CTGTGCATCATATCTTCGACTTCGCCCCAATACATTCATATGATTCT
```

Original Sequence:

1: PcWRKY44-426-4.PLB-F.J006040610
   1044 bases
   38 .. 463

2: PcWRKY44-426-5.PLB-F.J006040611
   1054 bases
   37 .. 462
